# The answer at our fingertips: Volume status in cirrhosis determined by machine learning and pulse oximeter waveform

**DOI:** 10.14814/phy2.15223

**Published:** 2022-03-11

**Authors:** Nikhilesh R. Mazumder, Avidor Kazen, Andrew Carek, Mozziyar Etemadi, Josh Levitsky

**Affiliations:** ^1^ Division of Gastroenterology and Hepatology University of Michigan Ann Arbor Michigan USA; ^2^ Gastroenterology Section VA Ann Arbor Healthcare System Ann Arbor Michigan USA; ^3^ Cardiosense Inc Chicago Illinois USA; ^4^ McCormick School of Engineering Northwestern University Chicago Illinois USA; ^5^ Department of Anesthesia Northwestern University Feinberg School of Medicine Chicago Illinois USA; ^6^ Division of Gastroenterology and Hepatology Northwestern University Feinberg School of Medicine Chicago Illinois USA

**Keywords:** biomarkers, cirrhosis, machine learning, physiology

## Abstract

**Objective:**

The objective of our study was to determine if the waveform from a simple pulse oximeter‐like device could be used to accurately assess intravascular volume status in cirrhosis.

**Methods:**

Patients with cirrhosis underwent waveform recording as well as serum brain natriuretic peptide (BNP) on the day of their cardiac catheterization where invasive cardiac pressures were measured. Waveforms were processed to generate features for machine learning models in order to predict the filling pressures (regression) or to classify the patients as volume overloaded or not (defined as an LVEDP>15).

**Results:**

Nine of 26 patients (35%) had intravascular volume overload. Regression analysis using PPG features (*R* ^2^ = 0.66) was superior to BNP (R^2^ = 0.22). Linear discriminant analysis correctly classified patients with an accuracy of 78%, sensitivity of 60%, positive predictive value of 90%, and an AUROC of 0.87.

**Conclusions:**

Machine learning‐enhanced analysis of pulse ox waveforms can estimate intravascular volume overload with a higher accuracy than conventionally measured BNP.

## Graphical abstract

A major challenge in managing patients with cirrhosis is that it is difficult to dose treatments due to the lack of a metric for intravascular volume status. In this pilot study, we developed a model that predicted volume status using the changes to a fingertip photoplethysmography waveform during breath holding.

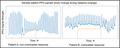

## 1 INTRODUCTION

The clinical course of patients with decompensated cirrhosis is commonly complicated by extravascular and intravascular volume overload related to portal hypertension and cardiomyopathy (Izzy et al., 2020). As a result of increased resistance to flow across the liver, multiple well‐described physiologic changes occur that result in increased portal pressures, splanchnic pooling of blood volume, and sympathetic activation due to effective arterial hypovolemia (Biggins et al., 2021; Oliver & Verna, 2010). Clinically, these changes manifest as ascites, the most common decompensating event in cirrhosis, but they also result in cardiovascular remodeling, renin‐angiotensin‐aldosterone activation, and symptomatic edema (D’amico et al., 2018; Izzy et al., 2020). Despite this complex hemodynamic interplay between the heart, liver, and kidney, the mainstay of treatment remains diuretic therapy (Biggins et al., 2021). Diuretics in decompensated cirrhosis provide relief indirectly, acting on intravascular volume, only affecting ascites and edema by increasing their resorption into the intravascular compartment. Unfortunately, if the rate of diuresis outpaces the rate of resorption then organ hypoperfusion can occur via intravascular depletion. Current diuretic dosing is often subjective, provider dependent, and based on trial‐and‐error strategies. Although direct measurement of intravascular volume is possible through cardiac catheterization, this is impractical for routine clinical care and current approaches to intravascular volume assessment center on physical examination. This approach is frequently inaccurate even when augmented with laboratory surrogates such as Brain Natriuretic Peptide (BNP), and as a result, adverse events such as hyponatremia, renal injury, and encephalopathy comprise the leading causes of the high hospitalization rates in this population (Stevenson, 1989; Volk et al., 2011). This problem is compounded by the rise of telemedicine in modern patient management making physical examination more difficult. Thus, a non‐invasive, easy to use, physiologic test correlating with intravascular volume status could improve patient safety by guiding treatment.

**FIGURE 1:**
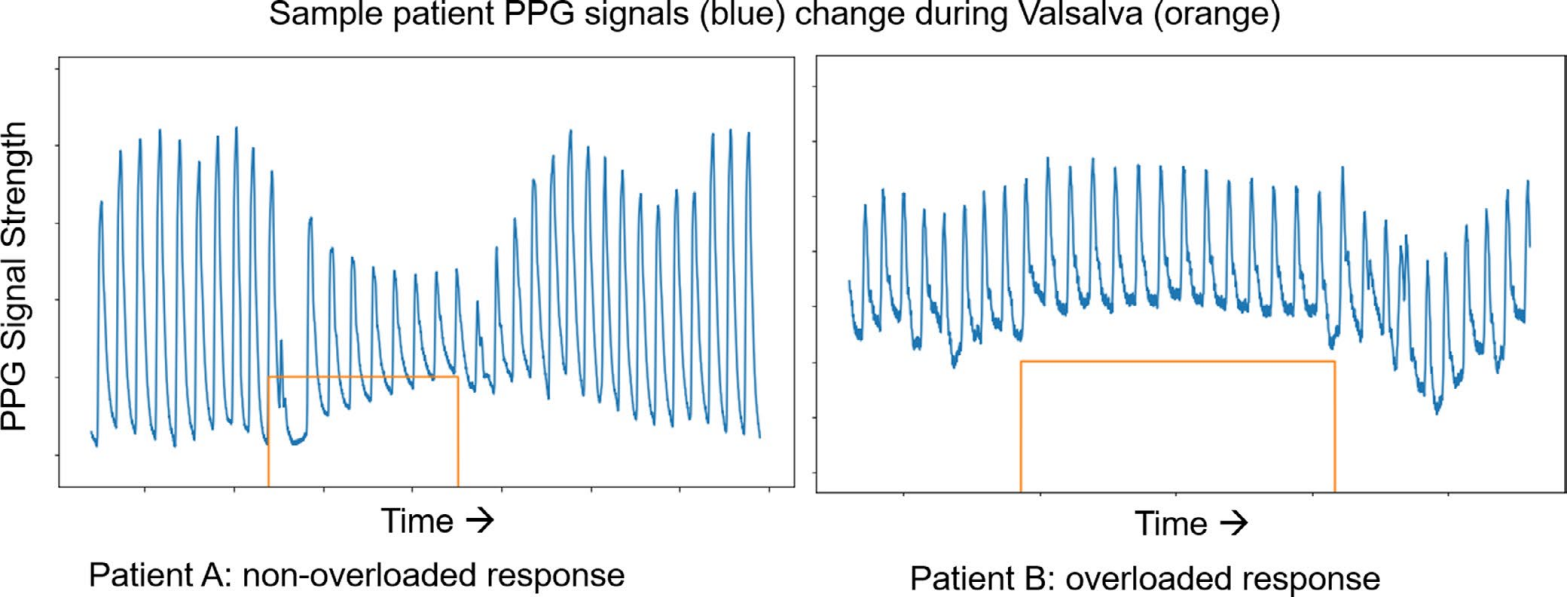
Sample PPG signal (blue) data from two representative patients. In patient A (Left), the introduction of breath holding (orange) decreases pulse amplitude which is a normal physiologic response. In patient B (Right) with intravascular volume overload, a similar stimulus has little to no effect on signal amplitude

Previous studies have shown that photoplethysmography (PPG) waveform changes, recorded by a pulse oximeter‐like device during Valsalva maneuver, highly correlate with volume status in systolic heart failure (Gilotra et al., 2017). Under normal physiology, cardiac filling should decrease at end‐Valsalva, an effect that is not observed in states of intravascular volume overload (Figure 1; Knowles et al., 1956). While prior studies have relied on quantifying a simple ratio between resting and end‐Valsalva waveform height as a surrogate of pulse pressure, newer machine learning techniques may provide opportunities for deeper analyses of waveforms. For instance, signal analysis of waveform shape has been used to infer other physiologic parameters such as systemic vascular resistance (Awad et al., 2001, 2007; Colquhoun et al., 2013; Takazawa et al., 1998), arterial stiffness (Chen et al., 2007; Imanaga et al., 1998; Park et al., 2019; Shelley et al., 1997), and continuous blood pressure (Millasseau et al., 2000; Xing & Sun, 2016). PPG signals have also been used to infer volume status in the setting of mechanical ventilation and surgery (Awad et al., 2007; Convertino & Sawka, 2018; Kim et al., 2017; Tusman et al., 2017). Unfortunately, these methods have not been applied to the question of intravascular volume assessment in patients with cirrhosis.

We therefore hypothesized that PPG signal changes during Valsalva could be used to accurately assess intravascular volume status in patients with cirrhosis.

## 2 METHODS

After IRB approval, we prospectively approached all cirrhotic patients undergoing non‐emergent left or right heart catheterization for any indication during the period of August 2018 through September 2019 at a large urban academic transplant center. Patients were excluded if they were unable to see the device, unable to follow commands, were admitted to intensive care, could not tolerate breath holding for 10 s, did not have pressures measured, or had atrial fibrillation at the time of testing. Otherwise, the decision to perform catheterization was determined by the primary medical and cardiology teams; the study had no input in this decision. On the day of catheterization and after informed consent, patients performed a Valsalva maneuver, as verified by an oral pressure transducer to 25 mmHg, for 10 s while in a seated position. Finger PPG waveforms were recorded before, during, and after Valsalva. Each patient underwent three trials with a 1‐min rest period. Patients also had blood draws for BNP on their catheterization day.

During catheterization, direct Left Ventricular End Diastolic Pressure (LVEDP) or Pulmonary Capillary Wedge Pressure (PCWP) measurements were recorded as part of planned, standard medical care. The research and cardiology teams were blinded to each other's results while performing testing.

The PPG and breath transducer device (Vixiar systems) transmitted waveform data for analysis. Covariates were collected and stored in a REDCap database. Waveforms were analyzed using Python and R software in order to measure and extract characteristics. Waveform feature generation was performed based on physiologic plausibility. In addition to generated features, age, interaction terms, and squared terms were also included as covariates for selection.

Two analyses were performed, regression and classification. Both analyses utilized a forward feature selection approach on the full dataset with a restriction to three features to reduce overfitting.

First, standard linear regression was used to predict the PCW or LV pressure based on PPG features. A Bland–Altman plot was generated for the final model. Model fit was assessed by calculating a coefficient of determination (*R* ^2^) after fivefold cross validation and comparing to a regression model with BNP as a sole feature.

For the classification analysis, “volume overload” was defined as a PCW or LV pressure >15 mmHg. Standard machine learning classification algorithms (linear discriminant analysis, logistic regression, K nearest neighbors, decision trees, and support vector machine with a radial basis function kernel) were assessed using the features selected in the regression analysis. Features were once again evaluated through an additional forward search. To avoid overestimation of model performance in the setting of unbalanced classes, classifiers were compared to a “dummy” classifier which simply defaulted to the most common, “non‐volume overloaded” class. Models were compared using accuracy, precision, recall, f1‐score, and area under the receiver operating curve (AUROC). A sensitivity analysis was performed for a cutoff of a PCW or LV pressure >10 mmHg as well.

## 3 RESULTS

Among 49 patients approached for consent, two patients were blind and five patients did not consent. Of 42 patients who consented, one patient could not complete the trial due to breathlessness, one patient had device issues, and one patient had her catheterization canceled. All remaining 39 patients completed PPG measurement and underwent catheterization as planned. At the discretion of the cardiology team, 26 patients underwent invasive cardiac pressure measurements, of whom 9 (35%) had intravascular volume overload.

Patient characteristics overall and stratified by intravascular volume overload status are listed in Table 1a. Patients were on average male (69%), 62.6 years old. The most common reason for liver disease was Non‐Alcoholic Steatohepatitis (NASH, 58%), followed by Alcohol (ETOH, 31%). Patients had significant liver disease, 50% of patients had a history of paracentesis and average Total Bilirubin of 3.9, International Normalized Ratio (INR) of 1.39, and Creatinine of 1.70, including 15% of patients on dialysis, resulting in an average Model for End‐Stage Liver Disease – Sodium (MELD‐Na) of 17.5. There were no significant differences in these characteristics between the intravascularly overloaded and non‐overloaded groups.

**TABLE 1a:** Patient characteristics

	Overall	No intravascular overload	Intravascular overloaded	*p*
*n*	26	17	9	
Male	69%	65%	78%	0.512
Age (mean (SD))	62.6 (10.4)	61.4 (9.2)	65.0 (12.6)	0.405
NASH (%)	58%	53%	67%	0.52
ETOH (%)	31%	35%	22%	0.512
HCV (%)	8%	6%	11%	0.65
MELD‐Na (mean (SD))	17.5 (8.8)	18.6 (9.9)	15.4 (6.3)	0.396
Na (mmol/L, mean (SD))	136.27 (5.14)	136.12 (6.17)	136.56 (2.51)	0.841
Cr (mg/dl, mean (SD))	1.70 (1.82)	1.63 (1.90)	1.83 (1.76)	0.796
Currently on dialysis %	15%	12%	22%	0.502
Albumin (g/dl, mean (SD))	3.30 (0.71)	3.08 (0.71)	3.70 (0.53)	0.031
Platelets (K/µl, mean (SD))	121.59 (60.25)	113.25 (57.52)	137.33 (65.58)	0.343
Total Bilirubin (mg/dl, mean (SD))	3.92 (7.67)	5.12 (9.37)	1.79 (1.96)	0.307
ALT (U/L, mean (SD))	35.20 (32.21)	37.12 (36.86)	31.78 (23.33)	0.699
AST (U/L mean (SD))	53.12 (38.11)	54.25 (42.85)	51.11 (30.12)	0.848
INR (mean (SD))	1.39 (0.27)	1.45 (0.26)	1.27 (0.23)	0.089
BNP (pg/ml, mean (SD))	382.52 (1061.75)	147.00 (132.10)	765.25 (1708.96)	0.203
Troponin‐I (ng/ml, mean (SD))	0.02 (0.02)	0.01 (0.01)	0.03 (0.02)	0.08
History of Paracentesis (mean (SD))	50%	47%	56%	0.689

Cardiac characteristics of patients are found in Table 1b. Although nonsignificant, the volume overloaded group tended to have higher Left Atrial Volume Index (LAVI), lower Left Ventricular Stroke Volume (LVSV), lower Left Ventricular Ejection Fraction (LVEF), and lower Tricuspid Annular Plane Systolic Excursion (TAPSE). Patients had similar rates of right and left heart catheterization, with some patients undergoing simultaneous procedures. Invasively measured filling pressures were 23.67 mmHg versus 10.59 mmHg (*p* < 0.001) in the overloaded compared to the non‐overloaded group.

**TABLE 1b:** Cardiac parameters

Days between Echo and cath (mean (SD))	40.79 (74.27)	38.08 (84.26)	46.67 (52.52)	0.822
LAVI (ml/m^2^)	36.16 (13.35)	35.16 (11.86)	38.38 (17.55)	0.67
LVSV (ml)	70.24 (36.55)	77.25 (40.29)	53.40 (19.39)	0.231
LVEDVI (ml/m^2^)	43.05 (23.54)	44.75 (25.65)	36.83 (15.38)	0.625
LVEF (%)	61.58 (15.57)	65.08 (12.45)	54.00 (20.00)	0.155
TAPSE (mm)	22.56 (5.97)	23.19 (5.46)	21.28 (7.25)	0.539
RVSP (mmHg)	44.50 (20.97)	46.00 (23.47)	41.00 (17.32)	0.751
Method of catheterization				
RHC %	69%	76%	56%	0.29
LHC %	62%	59%	67%	0.71
Filling pressure (mmHg)	15.12 (7.59)	10.59 (3.02)	23.67 (6.00)	<0.001

Abbreviations: LAVI, Left Atrial Volume Index; LVEDVI, Left Ventricular End Diastolic Volume Index; LVEF, Left Ventricular Ejection Fraction; LVSV, Left Ventricular Stroke Volume; RVSP, Right Ventricular Systolic Pressure; TAPSE, Tricuspid Annular Plane Systolic Excursion.

**FIGURE 2:**
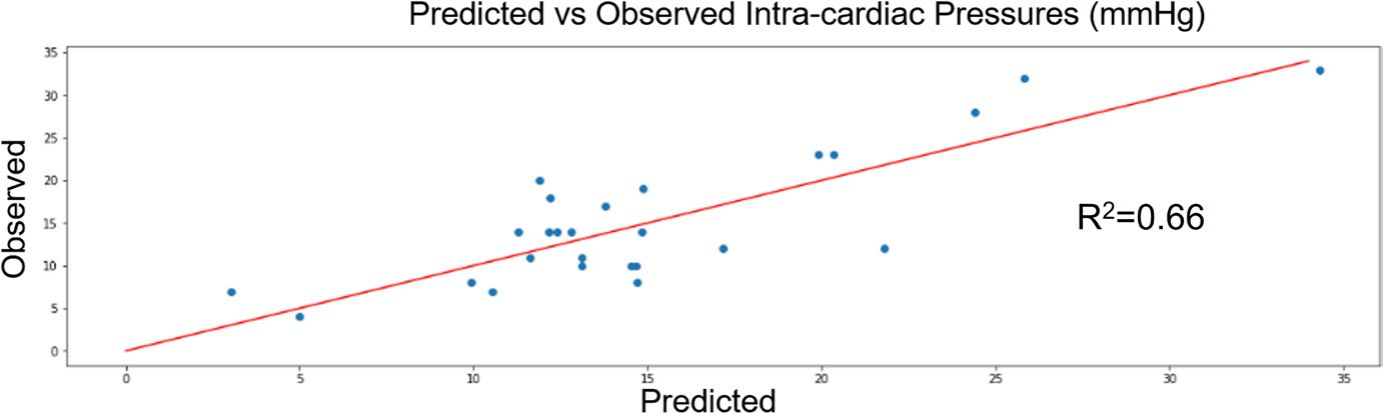
Results of the regression analysis. The trained machine learning algorithm can reliably predict intracardiac pressure based on waveform characteristics alone (*R* ^2^ = 0.66)

**FIGURE 3:**
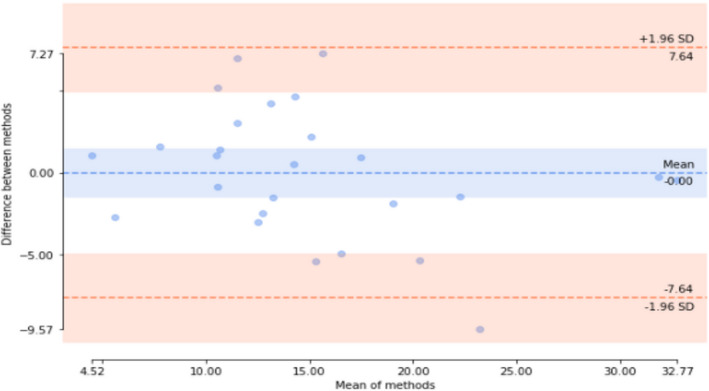
Bland–Altman plot. The predictions made in the regression analysis do not significantly change across the required pressure range

Results of regression and classification are noted in Table 2. Regression analysis using PPG waveform features alone resulted in a *R* ^2^ of 0.66 (Figure 2). This method correlated better with intravascular pressures than a model with BNP alone (*R* ^2^ = 0.22). A Bland–Altman plot demonstrated that predicted and actual intravascular pressures agreed across the range of measurement (Figure 3).

**TABLE 2:** Features of the final model and performance characteristics

Case	Terms	Results	Dummy results	BNP results
Regression	Interaction of the ratios of PPG RMS power and area‐under‐the‐curve at end rest and end Valsalva Interaction of the standard deviation of PPG amplitudes at end rest and subject's age Interaction of PPG amplitude at end Valsalva and ratio of area‐under‐the‐curve at end rest and end Valsalva	*R* ^2^: 0.73 Adj. *R* ^2^: 0.69 *p* < 0.001 CV *R* ^2^: 0.66	N/A	*R* ^2^: 0.25 Adj. *R* ^2^: 0.215 *p* = 0.0197 CV *R* ^2^: 0.23
Classification >10	Interaction of PPG amplitude and width at end rest Interaction of PPG amplitude at end Valsalva and subject's age Subject's age	CV accuracy: 74% CV Precision: 0.3 CV Recall: 0.3 CV F1: 0.27 CV AUROC: 0.77 CV Specificity: 0.93	CV accuracy: 70% CV Precision: 0.0 CV Recall: 0.0 CV F1: 0.0 CV AUROC: 0.5 CV Specificity: 1.0	CV accuracy: 71% CV Precision: 0.0 CV Recall: 0.0 CV F1: 0.0 CV AUROC: 0.6 CV Specificity: 0.93
Classification >15	Interaction of PPG width at end rest with the ratio of the PPG area‐under‐the‐curve at end rest and end Valsalva Interaction of PPG amplitude at end Valsalva and the standard deviation of PPG amplitudes at end Valsalva Interaction of the standard deviation of PPG amplitudes at end Valsalva and subject's age	CV Accuracy: 78% CV Precision: 0.9 CV Recall: 0.6 CV F1: 0.67 CV AUROC: 0.87 CV Specificity: 0.93	CV Accuracy: 61% CV Precision: 0.0 CV Recall: 0.0 CV F1: 0.0 CV AUROC: 0.5 CV Specificity: 1.0	CV accuracy: 57% CV Precision: 0.2 CV Recall: 0.1 CV F1: 0.13 CV AUROC: 0.65 CV Specificity: 1.0

Among the classification methods assessed, linear discriminant analysis (LDA) had the best performance. LDA was able to correctly categorize PPG waveforms in patients with volume overload with an accuracy of 78%. The sensitivity of this method was 60% with a positive predictive value of 90% and an f1 score of 0.67. The AUROC was 0.87. For comparison, the dummy classifier, which was set to “predict” a normal volume status for every subject, had an accuracy of only 61%.

When a different threshold of >10 mmHg was explored in a sensitivity analysis, the classifier above had an accuracy of 74% and AUROC of 0.77; however, this was only marginally better than the “dummy” classifier which had an accuracy of 71%.

## 4 DISCUSSION

Cirrhosis is a major cause of hospitalizations, morbidity, and mortality in the United States with nearly half of all patients dying within 2 years of diagnosis (D’amico et al., 2018; Serper et al., 2019). The chief cause of hospitalization and death stems from the complications of portal hypertension, the most common of which is ascites (D’amico et al., 2018). Diuretics are the most common treatment for this extravascular volume problem but are potentially dangerous because they must act through the intravascular compartment and no reliable method exists to measure this space. In this pilot study, our data demonstrate that fingertip PPG waveform analysis during breath holding using machine learning has promise in determining intravascular volume status in patients with cirrhosis. We were able to predict intravascular pressures more reliably than conventionally measured BNP or echocardiography. Typical of end‐stage liver disease, our patients had evidence of cardiomyopathy as well as renal dysfunction in addition to portal hypertension. Despite this, our model had high accuracy and excellent test characteristics, suggesting that the algorithm truly relies on physiology regardless of the combination of underlying organ dysfunction. This ability is highly desirable in the setting of end‐stage liver disease given the multisystem effects of the failing liver (Garcia‐Tsao et al., 2008; Møller & Henriksen, 2002). The potential for this technology is further bolstered by the fact that it is non‐invasive, easily repeatable, and can be automated.

Our results in this decompensated cirrhotic cohort add to the literature by combining concepts from engineering, physiology, and cardiology. For instance, PPG signal has been used to approximate arterial line tracing and infer hemodynamic parameters such as systemic vascular resistance in ICU settings (Awad et al., 2007; Colquhoun et al., 2013; Shelley et al., 1997; Tusman et al., 2017). In the setting of systolic heart failure and dialysis, basic ratios of PPG amplitude change during Valsalva have been correlated with intravascular pressures as measured by cardiac catheterization (Galiatsatos et al., 2015; Gilotra et al., 2017). PPG signal processing in real time has been utilized to sense sudden hemodynamic changes in the extreme setting of liver transplantation (Kim et al., 2017) and also failure of compensatory reserve in military trauma situations (Convertino & Sawka, 2018). Our study expands the literature by demonstrating the potential to apply the concepts of PPG signal processing to a less controlled situation—intravascular volume status management in cirrhosis.

While larger studies are needed to validate our findings, we hope that simple devices such as these will one day be used to provide accurate treatments based on physiology in patients with cirrhosis. Understanding when patients’ intravascular compartments are dangerously depleted could allow for home tracking of diuretics and potentially even allow for measured uptitration of diuretics which could reduce the frequency of paracentesis, emergency visits, and hospital admissions. Conversely, having a measure of when the intravascular compartment is replete could allow for early movement down the treatment algorithm of hepatorenal syndrome or sepsis where the first interventions are often to administer large amounts of volume (Biggins et al., 2021; Evans et al., 2021; Garcia‐Tsao et al., 2008). In fact, empiric administration of volume expanders without a sense of intravascular volume status may explain recent detrimental effects of albumin administration in hospitalized patients with cirrhosis (China et al., 2021) or the unexpected harm noted in terlipressin administered too late in type 1 hepatorenal syndrome (Wong et al., 2021).

Limitations of our pilot study stem primarily from small sample size. Due to this, feature selection could not be performed within each cross validation fold but was instead performed on the full dataset. Although this may introduce overfitting, we combatted this by limiting our model to only three features. Additionally, to train and assess our predictive models, we used a robust gold standard for intravascular volume status – intracardiac pressures measured immediately after our signal acquisition. Future studies should include a larger and more diverse patient population to ensure the generalizability of these results and determine the clinical utility of predicted pressures in the treatment of volume overload. Additionally, PPG may have applications in discerning other sought after parameters, such as the hepatic venous pressure gradient (HVPG), complications after Transjugular Intrahepatic Portosystemic Shunting (TIPS), or liver transplantation. Given that PPG is nearly universally available in modern medical care, these algorithms could be generalizable to multiple settings in order to enhance patient safety, improve outcomes, and reduce adverse events.

## CONFLICT OF INTEREST

AK and AC are founding members of Cardiosense Inc which uses machine learning to analyze medical signals for clinical use. None of the other authors have any conflicts of interest pertaining to this research project. This study was reviewed and approved by the the Northwestern Institutional Review Board (IRB) Office.

## AUTHOR CONTRIBUTIONS

Nikhilesh R. Mazumder participated in study concept and design; acquisition of data; analysis and interpretation of data; drafting of the manuscript; critical revision of the manuscript for important intellectual content; statistical analysis; obtained funding. Avidor Kazen contributed analysis and interpretation of data; drafting of the manuscript; critical revision of the manuscript for important intellectual content; statistical analysis. Andrew Carek contributed analysis and interpretation of data; drafting of the manuscript; critical revision of the manuscript for important intellectual content; statistical analysis; Mozziyar Etemadi participated in critical revision of the manuscript for important intellectual content; statistical analysis; study supervision. Josh Levitsky participated in study concept and design; analysis and interpretation of data; drafting of the manuscript; critical revision of the manuscript for important intellectual content; obtained funding.
